# Novel Treatments Targeting the Dysregulated Cell Signaling Pathway during Sepsis

**Published:** 2021-12

**Authors:** Justin H. Franco, Xiaohuan Chen, Zhixing K. Pan

**Affiliations:** Department of Medical Microbiology and Immunology, University of Toledo College of Medicine and Life Sciences, Toledo, Oh 43614 USA

**Keywords:** Sepsis, Septic Shock, Antioxidant, Retinoic Acid, NF-κB, IL-37

## Abstract

Previously characterized as a purely immune mediated disease, sepsis is now recognized as a dysregulated multisystem response against a pathogen. Recognition of the infectious agent by pathogen recognition receptors (PRRs) can initiate activation of the NF-κB signaling pathway and promote the secretion of proinflammatory cytokines. During sepsis, the activation of NF-κB is dysregulated and results in cytokine storm, or the pathologic release of cytokines. Current treatments for sepsis rely on broad spectrum antimicrobial medications and fluid replacement therapy, to neutralize the inciting pathogen and maintain adequate blood pressure. The addition of vasopressor therapy is also utilized when sepsis progresses to septic shock, which is defined by treatment resistant hypotension. Even though modern treatment guidelines have improved clinical outcomes, the mortality rate of sepsis and septic shock is still 15–20% and 20–50%, respectively. To reduce mortality, recent sepsis treatment research has focused on investigating novel therapeutics that can attenuate the dysregulated NF-κB signaling pathway. Antioxidants, such as Retinoic acid and Oxytocin, can reduce activation of the NF-κB pathway by neutralizing stimulatory reactive oxygen species (ROS). Likewise, anti-inflammatory agents can also affect the NF-κB pathway by decreasing the secretion of proinflammatory cytokines, such as TNFα and IL-6. Novel anti-inflammatory cytokines, such as IL-37 and IL-38, have recently been characterized and shown to reduce inflammation in mice with bacterial sepsis. Separately, antioxidants and anti-inflammatory cytokines show promise as potential therapies for sepsis, however, a combined therapy including both agents may prove more beneficial in further improving clinical outcomes.

## Introduction to Sepsis

The appearance of sepsis constitutes a medical emergency, requiring rapid medical intervention from healthcare professionals to prevent patient mortality. Previous characterizations of sepsis defined the disease as a Systemic Inflammatory Response Syndrome (SIRS) towards an underlying infectious agent [1–3]. The SIRS criteria for sepsis diagnosis focused on common physiological changes associated with infection, however, it didn’t take into account sepsis associated organ damage [2,3]. To better reflect the multifaceted nature of sepsis, recent studies define the disease as a dysregulated host response towards an infection that can progress to fatal organ dysfunction [2,3]. In a healthcare setting, sepsis is diagnosed using the Sequential Organ Failure Assessment (SOFA), which examines the health of six areas: namely respiration, coagulation, liver function, cardiovascular stability, central nervous system function, and renal activity [2]. Sepsis that is predominantly characterized by cardiovascular dysfunction, with drug resistant hypotension and elevated serum lactic acid, is categorized as septic shock [2,3].

The underlying infectious agent in sepsis can differ widely, exhibiting a bacterial, fungal, or viral etiology. Although the pathogenesis of sepsis depends on the identity of the offending organism, certain mechanisms are common among all types of sepsis [4]. Regardless of the infectious agent, sepsis can be divided into two distinct phases: a hyper-reactive and hypo-reactive immune response phase [4]. Recognition of the pathogen by the innate immune system can initiate a hyper-reactive immune response that results in an overwhelming release of proinflammatory cytokines, such as Tumor Necrosis Factor α (TNFα), Type I Interferons (IFNα/β), Interleukin 6 (IL-6), IL-8, and IFNγ [3,4]. The pathologic release of cytokines, known as cytokine storm, causes widespread endothelial dysfunction and coagulation, which can result in Multiple Organ Failure (MOF) [3,4]. TNFα in particular has been implicated as the key cytokine associated with the development of septic shock [5]. Following the hyper-reactive immune phase, sepsis becomes characterized by a hypo-reactive immune response that continues to affect the patient even after successful treatment of the inciting infection [3–5]. The immunosuppressive state predisposes patients to secondary infections that increases the 5 year mortality rate to 75%, even though current treatment guidelines have reduced the acute mortality rate of sepsis to 15–20% [5]. In cases were sepsis progresses to septic shock, the mortality rate increases substantially, with rates ranging from 20–50% [3].

Along with the relatively high mortality rate, sepsis is also a serious financial burden on local health care systems. In the United States (US) alone, hospital costs for sepsis treatment accounted for over $20 billion in 2011 [2]. The prevalence of sepsis in the US is approximated to be 1,000,000 cases annually, with an associated 200,000 deaths [5]. Global estimates of sepsis using epidemiological data from high income countries suggests that sepsis affects 31.5 million people annually, resulting in 5.3 million deaths [3,6].

Common risk factors associated with sepsis include immunosuppression, chronic infections, serious illness, physical trauma, and young (<2 years) or advanced (>55 years) age [3]. Additionally, sepsis represents a major problem in hospital Intensive Care Units (ICUs), were it is the primary cause of death [7]. Underlying comorbid conditions are associated with an increased possibility of infection in the ICU, especially cancer, chronic pulmonary disease, and Diabetes Mellitus [8]. Cancer in particular represents a significant risk factor for sepsis, with 6.4% of cancer patients developing sepsis within one year of diagnosis [9]. The prevalence of sepsis in pediatric ICUS (PICUs) ranges from 1–27%, with large variations existing between PICUs in developed versus developing countries [10].

Most sepsis cases are the result of an underlying bacterial infection, followed by fungal and viral infections. Bacterial sepsis represents over 60% of the total number of reported sepsis cases, resulting from either a polymicrobial, Gram-positive, or Gram-negative bacterial infection [4,11]. In the ICU setting, the predominant inciting bacterial pathogen for sepsis includes *Staphylococcus aureus*, *Enterococcus*, *Pseudomonas*, and *Escherichia coli* [11]. The second most prevalent cause of sepsis is from a fungal infection, accounting for about 20% of the total number of reported sepsis cases.^4,11^ Common causes of fungal sepsis include *Candida* and *Aspergillus* [4,11]. Unlike bacterial sepsis, fungal sepsis is most often acquired in the hospital, with 93% of diagnosed bloodstream candidiasis resulting from hospital acquired infection [4]. Viral infections are not thought to be significant contributors of sepsis when compared to bacterial and fungal pathogens, however, about 42% of septic patients are culture negative [12]. Although viral sepsis is rarely diagnosed in the clinical setting, the high percentage of negative cultures may suggest a greater role for viral pathogens in the development of sepsis [12]. Likewise, viral sepsis has emerged as a serious complication of severe COVID-19, with 76% of septic COVID-19 patients exhibiting a pure viral sepsis without evidence of a bacterial or fungal coinfection [13].

## Dysregulated Cellular Signaling during Sepsis

**Figure 1:**
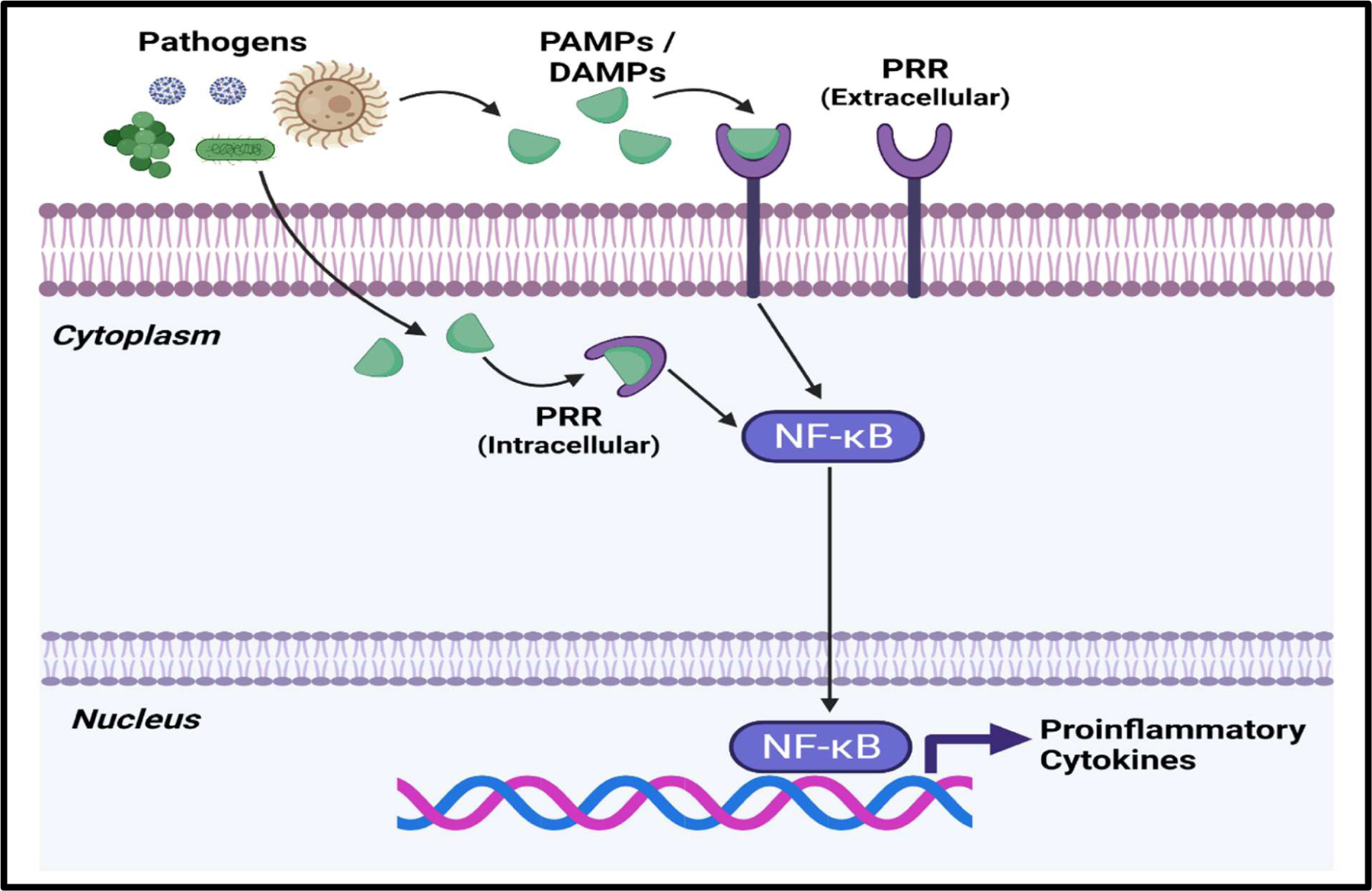
Overview of NF-κB Signaling Pathway during sepsis. Recognition of PAMPs and DAMPs by PRRs results in the activation of NF-κB, which facilitates the transcription of genes associated with Proinflammatory cytokines. **Abbreviations:** PAMPs: Pathogen Associated Molecular Patterns; DAMPs: Damage Associated Molecular Patterns; PRRs: Pathogen Recognition Receptors: NF-κB: Nuclear Factor κ B. This figure was created with BioRender.com

Although sepsis is a multifaceted disease process that can affect multiple organ systems, all variations of sepsis are initiated by the innate immune system [3,14–17]. Pathogen recognition receptors (PRRs) present on immune cells first recognize pathogen associated molecular patterns (PAMPs) or damage associated molecular patterns (DAMPs) [3,14–17]. The predominant types of PRRs utilized by host cells include toll-like receptors (TLRs), C-type lectin receptors, RIG-I like receptors, and NOD-like receptors [14]. In the case of Gram-negative bacterial sepsis, TLR-4 plays a major role in sepsis onset by recognizing bacterial lipopolysaccharide (LPS) [16,17]. Stimulation of PRRs results in activation of the NF-κB signaling pathway, leading to the production of proinflammatory cytokines (Figure 1) [3,14–17].

Under normal physiological mechanisms, the production of proinflammatory cytokines is self-limited and concludes with clearance of the inciting pathogen. During sepsis the activation of PRRs is exaggerated, resulting in excessive inflammation that can cause unintended tissue damage [16]. In particular, inflammation induced damage of endothelial cells can cause Disseminated Intravascular Coagulation (DIC) and increase the risk of MOF [14,15]. The hypercoagulable state exhibited during sepsis results from the disruption of endothelial cells, which release tissue factor that is responsible for initiating the coagulation cascade [14]. In addition to activating the coagulation system, the release of proinflammatory cytokines also diminishes the effect of compensatory anti-coagulative pathways. For instance, the increased expression of TNFα and IL-1β during sepsis greatly reduces the level of serum Protein C, which has anti-inflammatory and anticoagulative properties [14]. Reductions in Protein C, along with increased secretion of Plasminogen activator inhibitor type 1 (PAI-1), result in prolonged activation of the coagulation cascade and a diminished capacity to initiate fibrinolysis [14].

Disruptions in cellular signaling continue during the hypo-reactive immune phase of sepsis, which is characterized by immunosuppression [14]. The proinflammatory environment seen during acute sepsis becomes characterized by depressed immune function resulting from widespread immune cell depletion [14,15,18,19]. Sepsis induced apoptosis of CD4^+^ T, CD8^+^ T, B, and dendritic cells in lymphoid tissues, especially in the spleen, greatly increases the risk of developing a lethal secondary infection [14,15,18]. Widespread depletion of immune cells during sepsis is initiated by dysregulated activation of apoptotic pathways, mediated by the Fas-associated death domain (FADD) or the Mitochondria [18,19]. The remaining immune cells that don’t undergo apoptosis exhibit reduced inflammatory function, with splenic T cells demonstrating less capacity to produce TNFα and IFNγ [15].

## Current Treatments for Sepsis

Standard clinical guidelines for sepsis management focus on treating the underlying infectious agent and preventing organ failure. Upon diagnosis of sepsis, antimicrobials are given intravenously to eliminate the inciting pathogen [20–22]. Because bacterial infection is the most common cause of sepsis, broad spectrum antibiotics are often administered before the responsible pathogen has been identified by culture analysis [20–22]. Prompt administration of antimicrobial medications is especially important during septic shock, where studies have shown that each hour of therapeutic delay is associated with a decrease in survival of about 8% [21,23]. If the culture analysis provides evidence of an underlying fungal or viral infection, then antibiotic therapy is exchanged for effective antifungal or antiviral therapeutics [20–23].

The risk of organ failure during sepsis is due to inflammation induced vascular instability, which presents as hypotension or decreased blood pressure. Hypotension seen during sepsis is mitigated with aggressive fluid therapy, using crystalloid solutions to maintain organ perfusion [21]. During instances were fluid therapy is insufficient to maintain proper blood pressure, vasopressor therapy must be initiated to prevent subsequent organ failure [21]. Common vasopressors used to reestablish vascular stability include Norepinephrine and Dopamine, which promotes the contraction of vascular smooth muscle and increases cardiac output, respectively [21,24].

## Novel Treatments under Investigation

Modern guidelines for sepsis treatment focus on antimicrobial and vasopressor therapy to achieve the dual objective of eliminating the inciting pathogen and preventing organ failure. Although current treatment practices have improved patient outcomes, the associated mortality rate of sepsis and septic shock is still relatively high, ranging from 15–20% and 20–50%, respectively [3,5]. To improve survival rates from sepsis, novel therapeutics targeting the dysregulated cellular response have been investigated.

To target the hyper-reactive immune response seen during the initial phase of sepsis, various anti-inflammatory agents have been studied. Of note, Corticosteroids have been examined in patients with sepsis and septic shock due to their potent immunosuppressive properties and ability to increase blood pressure [21,25,26]. Administration of Corticosteroids, such as hydrocortisone, in patients with sepsis has failed to exhibit significant survival benefits, with a meta-analysis showing only a 2% reduction in the relative mortality rate [25]. As a result, the use of corticosteroids is only recommended for patients with septic shock that are unresponsive to fluid or vasopressor therapy [21]. Recent single center cohort studies combining corticosteroids with Thiamine and antioxidants, like Ascorbic acid (Vitamin C), have shown significant decreases in mortality, however, randomized clinical trials are needed to validate its efficacy [26].

**Figure 2:**
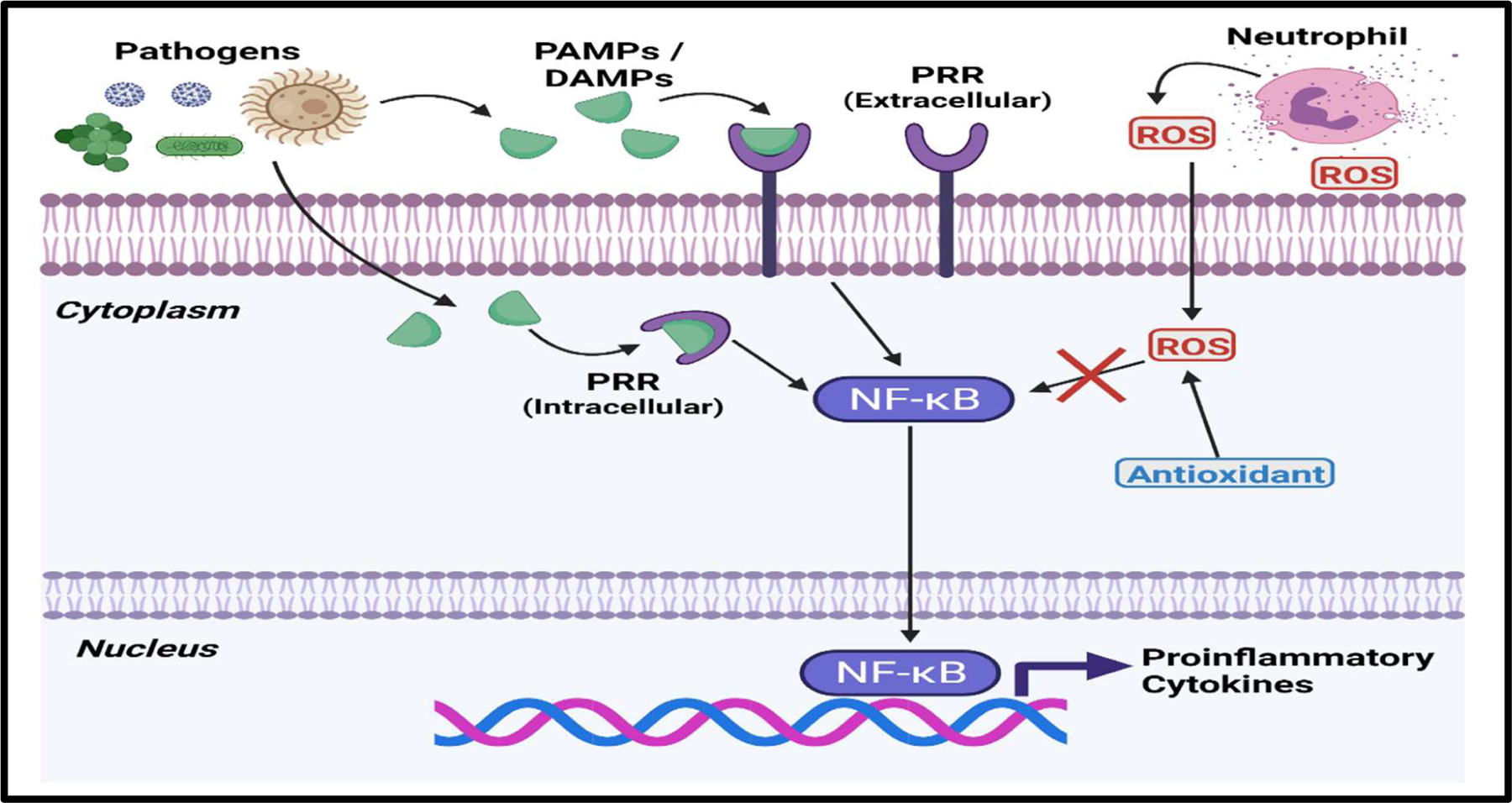
Antioxidants can negate ROS activation of NF-κB. ROS released from stimulated Neutrophils can induce the activation of the NF-κB signaling pathway, thus facilitating the production of proinflammatory cytokines. **Abbreviations:** ROS: Reactive Oxygen species. This figure was created with BioRender.com

By diminishing the activity of Reactive Oxygen species (ROS) that are secreted from immune cells in response to pathogens, antioxidants can potentially downregulate the NF-κB pathway (Figure 2). Dysregulated activation of NF-κB from ROS can result in cytokine storm and the overproduction of nitric oxide, which leads to systemic inflammation and mitochondrial dysfunction [27]. Common antioxidants such as Vitamins A, C, D, Edaravone, reduced Glutathione, Melatonin, and Oxytocin have been extensively studied in different models of sepsis [28–31]. Both Retinoic acid (Vitamin A) and Oxytocin are of special interest because they have been shown to modulate the immune response [30–34]. Retinoic acid is capable of downregulating the expression of TNFα by binding to specific retinoic acid response elements (RARE) in the genome [34]. Likewise, retinoic acid treatment has recently been shown to reduce the proliferation of Myeloid derived suppressor cells (MDSCs) in cecal ligation and puncture (CLP) mouse models [35]. Reductions in TNFα may reduce the effects of the hyper-reactive immune response phase, while inhibiting MDSC proliferation should promote CD4^+^ function and prevent the hypo-reactive immune response phase of sepsis [34,35]. When tested in a CLP rat model, Oxytocin was shown to significantly increase the plasma total antioxidant capacity and decrease serum levels of TNFα [30]. Investigations of Oxytocin’s effect in humans has yielded similar results, with one study reporting that it lowered the amount of serum TNFα, IL-4, IL-6, and IFNγ inducible protein 10 (IP-10) in patients inoculated with LPS endotoxin [33].

The use of antibodies directed against proinflammatory cytokines has previously been studied with mixed results [36,37]. Prior characterizations of anti-TNFα therapy in patients with sepsis and septic shock have been inconclusive, however, a meta-analysis of the published literature reported that septic patients treated with monoclonal antibodies had increased rates of survival [36]. Monoclonal antibodies directed against the IL-6 receptor, such as Tocilizumab, have also shown anti-inflammatory effects when administered to LPS stimulated monocytes [37]. Although antibody therapy exhibits properties that may benefit in sepsis, current research has begun to focus on the use of endogenous anti-inflammatory mediators as a novel treatment for sepsis.

Because of its function as a potent regulator of inflammation, IL-10 has been extensively studied as a possible treatment for sepsis [38,39]. Prior studies have shown that IL-10 is capable of downregulating the expression of proinflammatory cytokines and chemokines, however, recent investigations call into question IL-10’s efficacy as a potential treatment for sepsis [38,39]. Although IL-10 can decrease the production of TNFα in peripheral blood monocytes, it has also been shown to interact with the S100A9 protein and facilitate the proliferation of MDSCs [40]. IL-10 induced stimulation of MDSCs can potentially exacerbate the hypo-reactive immune response phase of sepsis, so recent studies have focused on newly characterized anti-inflammatory cytokines, such as IL-37 and IL-38 [41–46].

**Figure 3:**
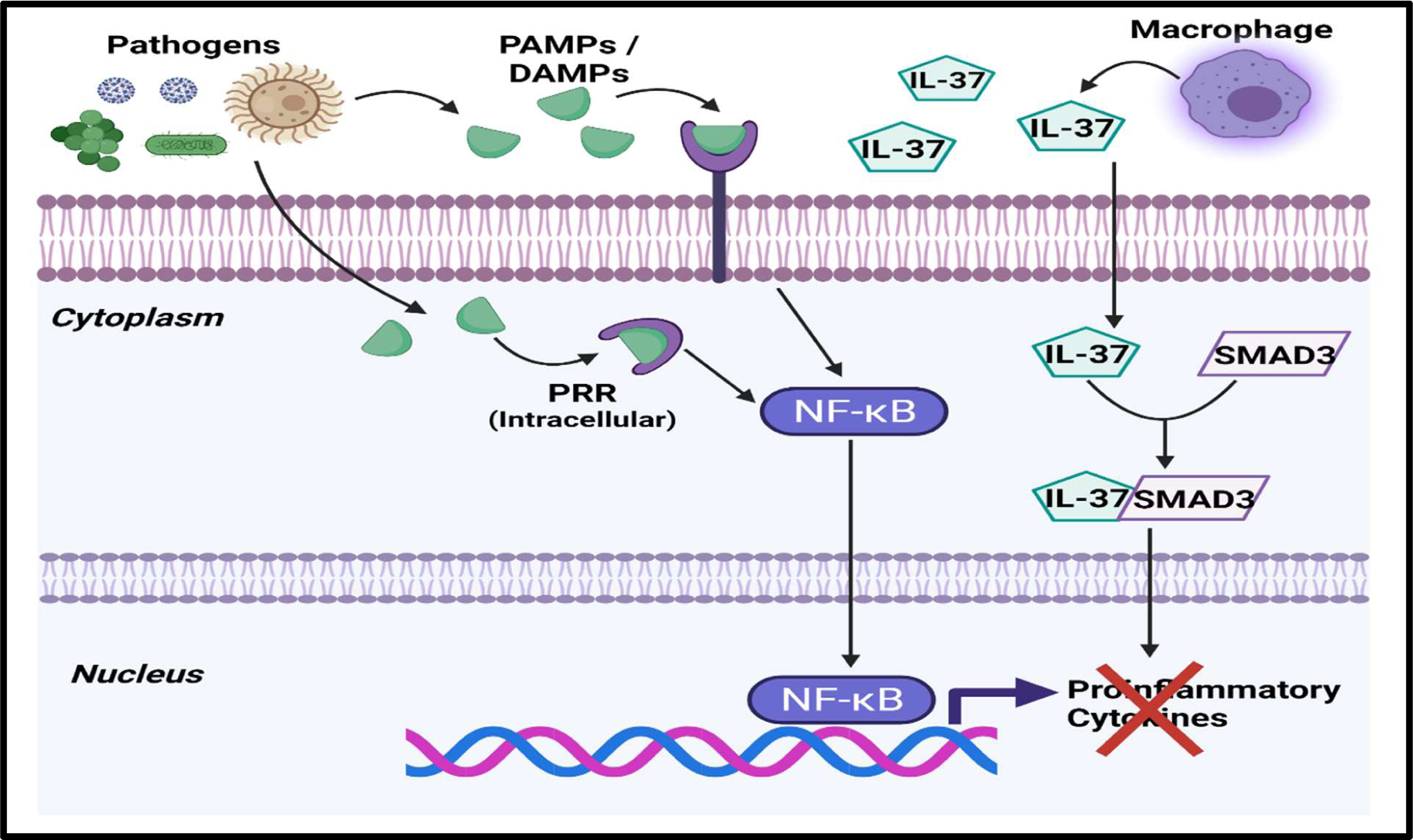
IL-37 can inhibit the expression of proinflammatory cytokines. When released from stimulated macrophages, IL-37 will bind to SMAD3 and downregulate the expression of proinflammatory cytokines. **Abbreviations:** IL-37: Interleukin 37; SMAD3: Small Mothers against Decapentaplegic family member 3. This figure was created with BioRender.com

IL-37and IL-38 are part of the IL-1 family of cytokines, which also includes the proinflammatory cytokines IL-1α, IL-1β, IL-18, IL-33, and IL-36 [38,41]. In vitro studies examining the effect of IL-37 in LPS stimulated monocytes revealed that it could reduce the production of TNFα, IL-6, and IL-8 [41]. When tested in LPS and CLP mouse models, both cytokines significantly reduced mortality from complications of sepsis, while IL-37 was also found to decrease sepsis associated cardiac inflammation [43–46]. The anti-inflammatory action of IL-37 results from its activity as a negative regulator of the NF-κB inflammation pathway and its action on dendritic cells [42]. Within the cytoplasm, IL-37 binds with SMAD3 to inhibit the expression of proinflammatory cytokines (Figure 3) [42]. When exposed to IL-37, dendritic cells (DCs) downregulate expression of major histocompatibility complex II (MHC II), which reduces their ability to stimulate Helper T cells [42]. Both IL-37 and IL-38 were also shown to increase the activity of CD4^+^CD25^+^ regulatory T cells (Tregs) under LPS stimulation [45,46].

## Conclusion

Established treatment guidelines for sepsis and septic shock focus exclusively on targeting the offending pathogen and maintaining vascular stability. As our understanding of sepsis has evolved, current research has been devoted towards testing novel therapeutics that can ameliorate the dysregulated NF-κB response characterized by sepsis. The dual antioxidant and immunomodulatory characteristics of Retinoic acid have made it a prime agent of study in animal sepsis models. Likewise, recent characterizations of IL-37 have shown it to possess potent anti-inflammatory properties in the context of sepsis. By reducing harmful ROS and promoting anti-inflammatory mechanisms, Retinoic acid and IL-37 may produce greater reductions in sepsis mortality if administered together versus if they were administered separately. Similar drug combinations have resulted in positive findings, such as the use of corticosteroids with Thiamine and Ascorbic acid. Further research examining the effect of combining antioxidants with anti-inflammatory cytokines is needed to determine if this therapeutic combination can improve patient outcomes in both sepsis and septic shock.
